# Crown Ether-Grafted Graphene Oxide-Based Materials—Synthesis, Characterization and Study of Lithium Adsorption from Complex Brine

**DOI:** 10.3390/ma17246269

**Published:** 2024-12-22

**Authors:** Ewa Knapik, Grzegorz Rotko, Marcin Piotrowski, Marta Marszałek

**Affiliations:** 1 AGH University of Krakow, Faculty of Drilling, Oil and Gas, al. Mickiewicza 30, 30-059 Krakow, Poland; 2 Cracow University of Technology, Faculty of Chemical Engineering and Technology, Warszawska 24, 31-155 Krakow, Poland

**Keywords:** lithium recovery, oilfield brine, adsorption, distribution coefficient, crown ether, graphene oxide, polyvinyl alcohol–chitosan hydrogel

## Abstract

Direct lithium extraction from unconventional resources requires the development of effective adsorbents. Crown ether-containing materials have been reported as promising structures in terms of lithium selectivity, but data on adsorption in real, highly saline brines are scarce. Crown ether-grafted graphene oxides were synthesized using 2-hydroxymethyl-12-crown-4, hydroxy-dibenzo-14-crown-4 and epichlorohydrin as a source of anchoring groups. The obtained carbonaceous materials were used to prepare chitosan–polyvinyl alcohol composites. The prepared materials (and intermediate products) were characterized using FTIR, XRD, Raman spectroscopy and SEM-EDS methods. Adsorption tests were performed in a pure diluted LiCl solution ([Li] = 200 mg/kg) as well as in a real, highly saline oilfield brine ([Li] ≈ 220 mg/kg), and the distribution coefficients (K_d_) were determined. The obtained results show that K_d_ in pure LiCl solution was in the range of 0.9–75.6, while in brine it was in the range of 0.2–2.3. The study indicates that the high affinity for lithium in pure LiCl solution is mostly associated with the non-selective interaction of lithium ions with the graphene oxide matrix (COOH groups). It was also shown that the application of dibenzo-14-crown-4 moiety to graphene oxide modification groups increases the affinity of the composite material for lithium ions compared to an analogous material containing 12-crown-4-ether groups.

## 1. Introduction

The transition towards a more sustainable economy relying on renewable energy sources requires a shift in raw material production [1]. The International Energy Agency estimates that the demand for oil will slowly decrease between 2025 and 2035, after which it will likely flatten with an average annual decline of 0.5% over the 2035–2050 period [2]. At the same time, the consumption of some minerals is expected to increase rapidly. Clean energy applications are stimulating the growth of the lithium-ion battery market [3]. According to the literature, global demand for lithium (Li) will soar by about 30% per year in the coming years and will reach 531 kt by 2030 (compared to 165 kt in 2023). The expansion of electric vehicles will entail a demand for lithium of approx. 1326 kt in 2040 [4] and up to 4.5 Mt in 2100 [5]. Regardless of the forecasts discussed above, lithium already has many applications, such as in batteries powering small electronic devices (smartphones, laptops, tablets); in the production of lubricants, synthetic rubbers and pharmaceuticals; in lightweight alloys for aircraft; and in heat-resistant glass and ceramics [6,7].

According to market reports, about 60% of Li produced in 2023 was extracted from hard rock deposits in open-pit mining operations [8,9]. The processing of the spodumene-bearing ore includes excavation, grinding, milling, froth flotation and refining of the obtained concentrate [10,11]. All operations are energy-intensive and lead to the generation of solid waste and CO_2_ emissions. This conventional lithium extraction is facing growing opposition from local communities; a good example is the Barroso Mine in northern Portugal, the development of which is being significantly hindered [12]. Lithium production from brine resources using an evaporation method also raises environmental and social concerns. In arid regions like the Tibetan Plateau [13] and the Atacama Desert [14], Li-rich continental brines are pumped into large, shallow open-air ponds, where about 90% of the original volume is evaporated due to wind and solar irradiation. The obtained concentrate with a Li content of about 6 g/L is transported to processing plants, where some impurities like boron, magnesium and calcium are removed, and lithium carbonate is precipitated using soda ash. The purity of the final product should be higher than 99.5 wt% to meet the requirements for Li-battery applications. Typically, to achieve this quality, a multi-step recrystallization technique is applied [15], resulting in a consumption of fresh water of up to 50 m^3^ per ton of final battery-grade Li_2_CO_3_ [16]. The disturbance of local hydrological relations [17] and the exposing of sensitive wetlands to harm [18] are the main objections raised by environmentalists regarding the exploitation of salars. The practical limitation of the evaporation method is the duration of the process, which can take up to 24 months and is strongly dependent on weather conditions [5].

There is a significant gap between the estimated potential demand for lithium and current production capacity. It is estimated that the development stage of a new lithium mining project usually takes around 10 years to become fully commercially operational. According to a report [19], to ensure the security of the lithium supply in the USA and Europe, lithium production from unconventional resources must be developed. For this reason, increasing attention is being paid to the recovery of lithium from geothermal waters [20,21], oilfield brines [6] and waste brine rejected from desalination plants [22]. There are numerous challenges to providing sufficient amounts of lithium in a sustainable manner [23]. New direct lithium extraction (DLE) methods are being intensively developed to minimize the environmental footprint of lithium production. In general, all DLE approaches aim to obtain Li-rich concentrates from low-grade brines without any evaporation stage using specific separations techniques, including membranes, sorbents and organic solvents [24,25,26].

Adsorption is often recommended as the most feasible method for lithium extraction due to its low cost, simple design, easy operation, flexibility and high efficiency [27]. Various sorbents have been tested so far, including zeolites [28], LiAl-layered double hydroxides [29], lithium manganese oxides (LMOs) [5], lithium titanium oxides (LTOs) [30] and many others [31,32]. Adsorbents are usually characterized in terms of their chemical stability, adsorption capacity, selectivity towards lithium ions and reusability. LMO ion sieves are known for their good adsorption kinetics and complex adsorption mechanism (both ion exchange and redox phenomena may occur) [33]. The adsorption capacity of LMOs depends on their synthesis conditions and varies from approx. 2 to 50 mg/g [5,34]. LTOs achieve adsorption capacities similar to LMOs (8–59 mg/g [5]) but are more chemically stable (no dissolution during regeneration).

A comprehensive review of sorbents and other methods for lithium recovery conducted by Khalil et al. [5] shows that membrane-like composite materials based on graphene oxide (GO) modified with crown ether offer the highest adsorption capacity (168.5 mg/g), and their adsorption properties are 2–3 times better than those of inorganic sorbents. The result is impressive compared to other sorbents, but only one article about this type of material has reported such a high adsorption capacity [35]. Hence, we decided to explore this area.

Crown ethers are cyclic polyethers with a regular structure containing several ether groups (R−O−R’). These compounds can form host–guest complexes with different cations, allowing selective separation of targeted ions. The size of the central cavity in the crown ether ring determines the size of the cation it can coordinate [36]. Therefore, for example, 18-crown-6 exhibits a high affinity for K^+^, 15-crown-5 for Na^+^, and 12-crown-4 for Li^+^ [37]. The crown ethers themselves occur in liquid form and are rather expensive (in the order of several tens of dollars per 1 g), so they are not directly used for lithium extraction, but may be used as modifiers/active substances immobilized on the surface of membranes or sorbents.

GO seems to be a perfect carrier for crown ethers due to its large surface area and unique surface structure, which contains different oxygen functional groups (hydroxyl, epoxy, and carboxylic acid groups) which are relatively easy to modify [38]. GO-based membranes are reported to have various applications [39], mainly in the removal of contaminants from wastewater [40,41,42].

Baudino et al. [43] tested GO functionalized with a 12-crown-4-ether using a polycarbonate support membrane for lithium extraction in a dead-end pressure filtration setup. Their results show that the recovery of lithium from diluted solutions (initial Li concentration of 7 mg/L) is still challenging, with an average lithium recovery of 70% and lithium uptake of 5 mg/g in each filtration cycle. A higher adsorption capacity of 10.45 mg/g was obtained by Cui et al. [44] for a PVDF/GO hybrid membrane with a polydopamine coating layer using a pure LiCl solution (100 mg Li/L) after 3 h of contact. Zheng et al. [35] reported an adsorption capacity of 97.23 mg Li/g for a nanofiber membrane consisting of functionalized graphene oxide, chitosan and polyvinyl alcohol, but the system was tested under equilibrium conditions using a concentrated LiCl solution (1000 mg/L). Due to the limitations of the above-mentioned results, we decided to study such PVA–chitosan composites, including tests in real brine.

The density functional theory (DFT) simulations performed by Abdulazeez [45] suggest that graphene sheets modified with crown ethers may exhibit poor selectivity towards lithium ions in more complex solutions. A comprehensive review by Zavahir et al. [46] on ion-imprinted crown ether-based membranes clearly shows that research devoted to these membranes is at a very early stage and detailed laboratory verification is needed. The reported results are promising, but the systems tested so far have used only synthetic solutions, the compositions of which correspond poorly to such complex raw materials as geothermal waters or reservoir brines. Thus, there is a significant research gap that limits the commercialization of these materials.

In this study, we tested a membrane-like material consisting of GO functionalized with crown ethers, using both model (200 mg/kg lithium chloride solution) and real brine sampled from a Polish oil field. The reservoir brine contained about 200 mg/kg of lithium and 1000 mg/kg of Mg; its salinity was very high, up to 250 g/kg, making it a promising but difficult raw material. To the best of our knowledge, this is the first study to have tested this type of material in real highly saline brine. The modification of the crown ether-grafted graphene oxide-based material was carried out using commercially available 2-Hydroxymethyl-12-crown-4, previously reported in the literature, and hydroxy-dibenzo-14-crown-4, a compound that has not been employed for this purpose to date. Furthermore, intermediate products of the modification (such as GO and GO functionalized with epichlorohydrin) were characterized, and their affinity for lithium ions was investigated.

## 2. Materials and Methods

### 2.1. Materials and Chemicals for Synthesis

Analytical-grade chemicals were used to synthesize the adsorbents, unless otherwise indicated. Graphite flakes (325 mesh particle size), acetonitrile, 1,3-dibromopropane, 2-Hydroxymethyl-12-crown-4, PVA (M_w_ 13,000–23,000, 98% hydrolyzed), chitosan (degree of deacetylation > 95%, low molecular mass, viscosity of 1% in 1% of acetic acid equal to 20–300 mPa∙s), sodium hydride (60% dispersion in mineral oil) were delivered by Sigma Aldrich (St. Louis, MO, USA). Ethanol (96%), acetone, KMnO_4_, H_2_SO_4_ (98%), H_3_PO_4_ (85%), H_2_O_2_ (30%), HCl (35–38%), NaOH, epichlorohydrin, catechol, lithium hydroxide monohydrate, CaH_2_, N,N-dimethylformamide (DMF), acetic acid (99.5%), dichloromethane were purchased from Pol Aura (Morąg, Poland).

Anhydrous acetonitrile was dried using CaH_2_ and distilled from the above CaH_2_ prior to the reaction. Anhydrous lithium hydroxide was prepared from the monohydrate by drying at 150 °C until the appropriate mass was achieved.

In the adsorption studies, a pure lithium chloride solution with a lithium concentration of 200 mg/kg and real brine were used. The lithium chloride solution was prepared by dissolving an appropriate amount of anhydrous lithium chloride (Sigma Aldrich, St. Louis, MO, USA) in deionized (DI) water. The real brine used for this study was sampled from an oilfield located in the Polish Lowlands. The brine was collected from a central storage tank where water from different wells/zones is mixed together, resulting in an averaged composition of water sample. The obtained water is a chloride-type brine with the following major cations content: 7.21 wt% of Na, 3.0 wt% of Ca, 1000 mg/kg of Mg and 220 mg/kg of Li. Since no oil was visible in the water, it was used for further experiments as received (without any treatment).

Deionized water from the Millipore system (Merck KGaA, Darmstadt, Germany) (>18 MΩ) was used to dilute samples to a level suitable for measurements, as well as for all synthetic procedures.

### 2.2. Synthesis of Graphene Oxide

Synthesis of graphene oxide was performed according to the so-called improved method [47,47], with some changes described below.

Exfoliation of the reaction product was carried out by repeating the freeze/thawing process [48] seven times. The dispersion in water obtained as a product was immediately used in the next reaction step. Samples for material characterization and adsorption tests were additionally washed with ethanol (2 times) and acetone (2 times) and dried under vacuum at 40 °C for 4 h. The detailed synthesis procedure is as follows: KMnO_4_ (18 g) was slowly added (over 3 h) in small portions to a mixture of concentrated H_2_SO_4_ and H_3_PO_4_ in the ratio of 9/1 (360/40 mL) and graphite flakes (3 g). The mixture was then heated to 50 °C and stirred for 12 h. After this time, the reaction mixture was cooled to room temperature and poured onto ice (400 mL) containing 30% H_2_O_2_ (3 mL). A yellow suspension was obtained, in which no black graphite particles were visible. Next, the suspension was filtered through a sieve (50 μm) to remove unreacted particles. The resulting filtrate was centrifuged (3500 rpm for 10 min), and the supernatant was decanted. The obtained solid material was washed with 200 mL of DI water, 200 mL of 30% HCl and again with 200 mL of DI water (3 times). Centrifugation (3500 rpm for 10 min) was applied to recover the solid material. The resulting slurry was redispersed in 1.7 L of DI water using an ultrasonic bath (room temperature, 30 min, 130 W) and quickly frozen. The samples were then thawed. Seven freeze/thawing cycles were carried out. The obtained dispersion in water (pH approx. 3) was subsequently reacted with epichlorohydrin, and a small amount (1/5) of the dispersion was collected as material for the sample characterization, adsorption test and GO-Epi1 synthesis.

### 2.3. Synthesis of Epoxy-Functionalized Graphene Oxide (GO-Epi)

The synthesis was carried out using two procedures. GO-Epi1 was synthesized according to the procedure described in [35] (Procedure 1). The synthesis of GO-Epi2 was based on [49], with some changes (Procedure 2): 700 mL of epichlorohydrin was added over 8 h to 1.36 L of freshly prepared graphene oxide dispersion. During epichlorohydrin dosing, the pH of the reaction mixture was controlled and adjusted (using a 10% NaOH solution) to values in the range of 8.5–9.5. The concentration of epichlorohydrin was under the solubility level, which is approx. 70 g/L. The temperature of the mixture during the reaction was 60 °C. After adding all the epichlorohydrin, the reaction mixture was left at 60 °C overnight. The solid product was isolated by centrifugation at 4000 rpm for 30 min and washed three times with DI water (200 mL each portion), ethanol (2 times with 100 mL) and acetone (2 times with 100 mL). The collected solid was dried under vacuum (40 °C for 4 h).

### 2.4. Synthesis of Hydroxy-Dibenzo-14-Crown-4 (Hydroxy-DB14C4)

The synthesis was carried out according to [50], and the product of the reaction was used for GO-Epi functionalization without any additional purification. The identity of the reaction product was confirmed by ^1^H NMR and ^13^C NMR. Samples for spectroscopic analyses were purified using column chromatography. Moreover, the Fourier transform infrared (FTIR) spectrum was recorded.

### 2.5. Synthesis of Crown Ether-Grafted Graphene Oxide (GO-Epi-E)

Grafting of crown ethers was performed according to the protocol described in [35]. The reaction products with commercially available 2-Hydroxymethyl-12-crown-4 were described as GO-Epi1-E1 and GO-Epi2-E1 (two samples of epichlorohydrin-modified GO were used), while the reaction product with hydroxy-DB14C4 was described as GO-Epi2-E2.

### 2.6. Fabrication of Crown Ether-Grafted Graphene Oxide–Chitosan–Polyvinyl Alcohol Composite Material

The fabrication was performed according to the procedure reported in [35]. The addition of the graphene oxide-based materials was the same as in the cited publication. Descriptions of the obtained samples are given in Table 1.

### 2.7. Adsorption Studies

Adsorption tests were performed according to a previously published procedure under similar conditions [31]. Adsorption experiments were carried out using a pure LiCl solution with a lithium concentration of 200 mg/kg and real brine in a batch system. Approximately 1 g of adsorbent (or 1 g of the appropriate crown ether diluted with dichloromethane in a 1:1 (wt/wt) ratio) and 10 g of brine were placed in a plastic container, shaken vigorously for 1 h and allowed to settle for another 23 h (total contact time was 24 h). After this time, the mixture was centrifuged, and the supernatant was appropriately diluted to determine lithium concentration by atomic absorption spectrometry (AAS) measurements. Adsorption was tested at acidic reaction, i.e., pH = 5, and at a slightly alkaline reaction, i.e., pH = 8 (the pH was corrected with 1 M NaOH solution).

The values of the distribution coefficient (*K_d_*) of lithium ions were calculated according to Equation (1):

(1) $$K_{d}=\frac{\left(C_{0}-C_{e}\right)\cdot \left(\frac{m_{solution}}{m_{adsorbent}}\right)}{C_{e}}$$

where $C_{0}$ (mg/kg) is the initial concentration of lithium in lithium chloride solution or brine, $C_{e}$ (mg/kg) is the concentration of lithium at equilibrium, *m_solution_* (g) is the mass of lithium chloride solution or brine and *m_adsorbent_* (g) is the mass of adsorbent (crown ether) used in the adsorption experiment.

The values of recovery of lithium ions *R* (%) were calculated according to Equation (2):

(2) $$R=\frac{\left(C_{0}-C_{e}\right)}{C_{0}}\cdot 100\%$$

The operation adsorption capacity *q* (mg/g) was calculated according to Equation (3):

(3) $$q=\left(C_{0}-C_{e}\right)\cdot \left(\frac{m_{solution}}{m_{adsorbent}}\right)\cdot 1000$$

### 2.8. Analytical Methods

Scanning electron microscopy (SEM) images were obtained by detecting the secondary electrons at 5 kV accelerating voltage using an SU8000 field emission scanning electron microscope (Hitachi, Tokyo, Japan). All SEM images were taken at 5000× magnification. Energy dispersive X-ray spectroscopy (EDS) microanalysis was performed for three areas (400 μm^2^) of selected samples (the average was calculated). The analyses were performed at two acceleration voltages: 5 kV and 15 kV. The total acquisition time for analysis was 45 s.

Raman spectra were recorded using a WITec Alpha 300M+ spectrometer (WITec, Ulm, Germany) with 532 nm wavelength laser and 0.1 mW power. A 50× long working distance objective and 600 grooves/mm grating were used for measurements. Typically, 10 accumulations of 2 s scans were collected at each point. Three measurements were taken per sample and the result was averaged.

X-ray diffraction (XRD) patterns were recorded at a 2θ angle ranging from 5 to 60° using a Philips X’Pert powder diffractometer (PANalytical, Almelo, Netherlands) equipped with Cu K_α_ radiation source operating at 40 kV and 20 mA.

Fourier transform infrared spectra in the range of 500–4000 cm^−1^ were recorded using a Nicolet iS5 FTIR spectrometer equipped with an iD7 attenuated total reflection (ATR) accessory (Thermo Fisher Scientific, Waltham, MA, USA). The total number of interferograms (single scans) for each spectrum was 32.

The ^1^H NMR and ^13^C NMR spectra of synthesized crown ether (solution in CDCl_3_; concentration approx. 10 mg/mL) were recorded using a 500 MHz JNM-ECZR500 RS1 spectrometer (JEOL, Tokyo, Japan).

The concentration of lithium in the tested samples before and after adsorption was measured by the atomic absorption spectrometry method using the PerkinElmer AAnalyst 100 Spectrometer (PerkinElmer Inc., Waltham, MA, USA). Details of the method are described in [31].

## 3. Results and Discussion

### 3.1. Synthesis and Characterization of Crown Ether Grafted Graphene Oxide Based Materials

#### 3.1.1. Synthesis Remarks

**Figure 1:**
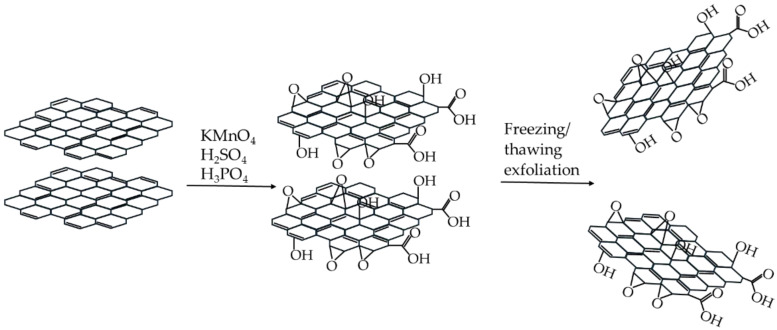
Reaction scheme for graphene oxide synthesis.

The reaction scheme for the synthesis of graphene oxide is presented in Figure 1. The so-called “improved method” for the synthesis of graphene oxide was applied because a more oxidized product is obtained compared to Hummers’ method. Such a product contains more reactive groups, which can be anchor groups for further functionalization. According to the literature [35], graphene oxide is a carrier for crown ethers, hence it was desirable to use a product containing a larger number of attachment groups. To support exfoliation, a freeze/thawing process [48] was used because it is easy to scale. The obtained dispersion in water was used directly in the reaction with epichlorohydrin, as we noticed that the properties of graphene oxide change with storage time (e.g., color, O/C ratio, dispersibility). Moreover, drying and subsequent dispersing also change the properties of GO and are impractical (two additional steps in the synthesis). Observations concerning the stability of GO during storage have also been reported in the literature [51], so we decided that the best option was to use a freshly prepared dispersion in reaction with epichlorohydrin.

**Figure 2:**
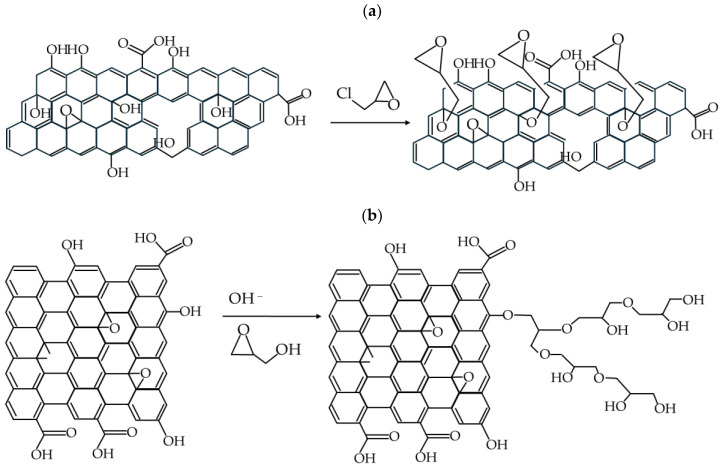
Scheme of graphene oxide modification with epichlorohydrin: (**a**) the main reaction postulated in the literature, (**b**) a side reaction leading to the formation of a polyether polymer.

Several procedures for functionalizing graphene oxide with epichlorohydrin have been published [35,49,52]. According to the literature data, the use of GO functionalized with epichlorohydrin is a convenient method for anchoring crown ethers on the surface of the material [35,49]. Usually, FTIR spectroscopy is used to control the quality of the reaction product, but the reported spectra of obtained material vary in previously published articles. It should be noted that the reaction product (GO-Epi) exbibits vibrations similar to the substrate (GO); these are associated with the epoxy rings and C-O-C and C-O moieties (see Figure 2a). Therefore, there is no clear range in FTIR spectra that corresponds to unambiguous information about the number of introduced epoxy groups. Furthermore, epichlorohydrin under basic conditions, most often used in the reaction, may also react with nucleophilic groups of GO through the epoxy ring opening. Additionally, the attached groups may still be reactive in the process, and some kind of branched polyether polymer may be formed [52] (see Figure 2b).

In summary, functionalization of epichlorohydrin can result in different structures depending on the reaction conditions, the materials used and other not fully controlled factors. For this reason, we made the decision to employ two procedures of functionalization. Procedure 1 (sample described as GO-Epi1) is strictly reproduced from [35], while Procedure 2 is based on [49] with a few changes: (1) epichlorohydrin was added into the reaction mixture in several portions; (2) the structure of the reaction product was monitored using FTIR spectroscopy; (3) the pH of the reaction mixture was controlled during the reaction. It is important to add epichlorohydrin in portions because epichlorohydrin undergoes relatively quick hydrolysis in an aqueous solution at pH > 8, so the efficiency of epichlorohydrin use may be low and a large excess of epichlorohydrin must be applied (e.g., according to [49], the ratio of epichlorohydrin to GO should be in the range of 100–300 g/g).

**Figure 3:**
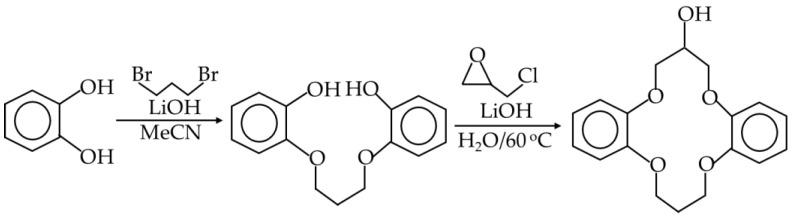
Reaction scheme for hydroxy-DB14C4 synthesis.

Commercially available 2-Hydroxymethyl-12-crown-4 was used as a reference crown ether in the modification of graphene oxide. It is a popular reagent for such modification [35]. However, from the study on lithium capture selectivity of various crown ethers, it is known that the 12-crown-4 ring is less selective in comparison to 14-crown-4 ethers. The selectivity coefficient of Li^+^/Na^+^ separation in model system is equal to 1.7 for the 12-crown-4 and 20 for the 14-crown-4 [53]. Based on the 14-crown-4 ring, many lithium selective crown ethers with anchoring groups have been developed [50,54]. According to the data presented in the literature and taking into account the simplicity and scalability of the synthesis, we decided to use hydroxy-DB14C4. The use of this ether is a compromise between ease of synthesis and selectivity. Preparation of the hydroxy-DB14C4 was carried out according to the published procedure [50] (see reaction scheme in Figure 3). The identity was checked spectroscopically. In addition, we recorded the FTIR spectrum for the crown ether, which is useful in assessing the effectiveness of graphene surface modification.

**Figure 4:**
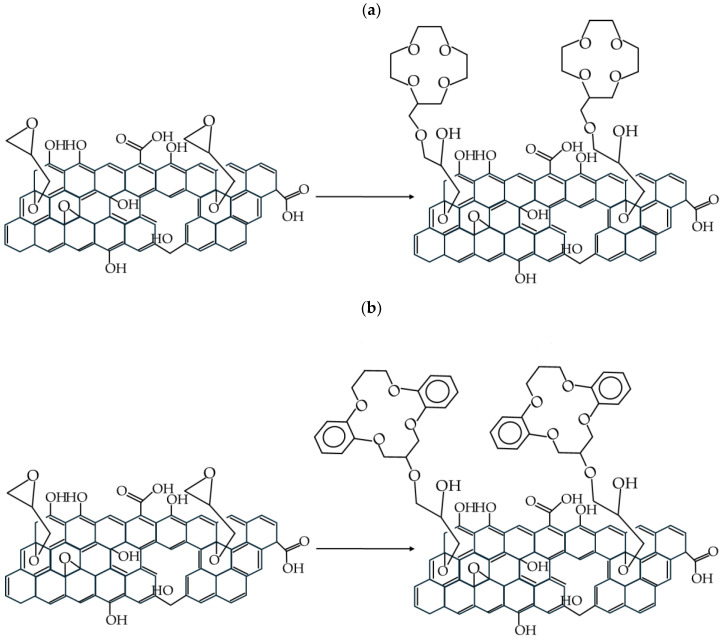
Reaction scheme for introduction of 12-crown-4 (**a**) and dibenzo-14-crown-4 (**b**) onto the modified GO.

To anchor the crown ethers on the graphene oxide surface, coupling of the hydroxy groups of the crown ethers with the epoxy groups of the functionalized graphene were used (see Figure 4). A procedure described in the literature [35] was used for this reaction. It should be noted that according to the literature [35], approx. 2.1 moles of NaH per 1 mole of crown ether were used. Thus, during the reaction with graphene oxide, an excess of NaH is present in the reaction mixture. Considering that NaH is not only a strong base (deprotonating agent) but also a reducing agent, partial reduction of graphene oxide may occur. We observed that the color of the reaction product changed from dark brown to black. Moreover, the usage of DMF as a solvent is limited only to a small scale, as NaH can also react with DMF; the reaction occurs with an exothermic effect and many gaseous products (such as dimethylamine) are released, so the reaction may be difficult to control on a large scale [55]. Nonetheless, we did not optimize this reaction step, as some specific conditions could have an impact on the final product. Further optimization of this process is necessary and will be undertaken in the future.

Two samples of graphene oxide modified with epichlorohydrin were used for synthesis with 2-Hydroxymethyl-12-crown-4. After preliminary lithium adsorption tests (*vide infra*), we observed that the sample prepared from GO-Epi2 exhibited slightly better properties, so we decided to prepare the hydroxy-DB14C4-grafted graphene oxide only with GO-Epi2.

The obtained crown ether-grafted graphene oxide materials were used for fabrication of chitosan–polyvinyl alcohol composite. The material was prepared according to the procedure published by Zheng et al. [35]. During the gel preparation, we observed that for crown ether-modified graphene oxides, it was difficult to disperse them evenly in Chit-PVA solution. The modified graphene oxides formed agglomerates visible to the naked eye. Therefore, the time for ultrasonication during the preparation was extended. At this point, it should be mentioned that the cited article [35] shows SEM images of the composite material, but it is difficult to see graphene oxide material that is evenly distributed. Thus, it is possible that graphene oxide is present in larger lumps that occur less frequently.

#### 3.1.2. Morphology Studies Using Scanning Electron Microscopy

**Figure 5:**
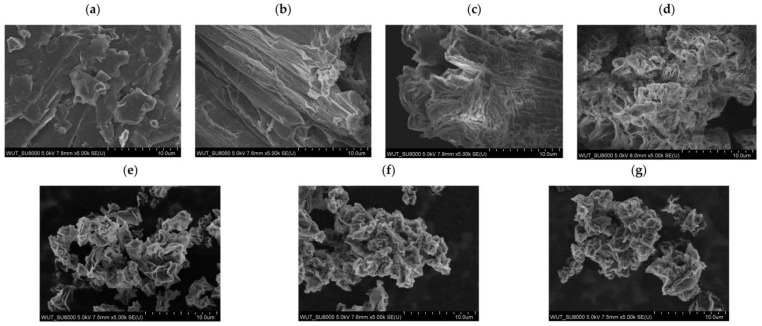
SEM images of (**a**) raw graphite; (**b**) graphene oxide; (**c**) GO modified with epichlorohydrin according to Procedure 1 (GO-Epi1); (**d**) GO modified with epichlorohydrin according to the Procedure 2 (GO-Epi2); (**e**) GO modified with epichlorohydrin and 2-hydroxymethyl-12-crown-4 according to Procedure 1 (GO-Epi1-E1); (**f**) GO modified with epichlorohydrin and 2-hydroxymethyl-12-crown-4 according to Procedure 2 (GO-Epi2-E1); (**g**) GO modified with epichlorohydrin and hydroxy-DB14C4 (GO-Epi2-E2).

The surface morphology of the carbonaceous materials was characterized using a scanning electron microscope after each synthesis stage. Figure 5 shows the SEM images starting from raw graphite powder (Figure 5a) up to graphene oxide functionalized with crown ethers (Figure 5d,f,g).

The original graphite exhibits a smooth and compact surface, with no inclusions or fibers, which suggests its homogeneous composition and high purity. Graphite occurs in its natural state in the form of flakes, and such thick tiles of irregular arrangement are visible here. Intensive oxidation in the presence of strong acids leads to the formation of graphene oxide and changes in textural properties. The exposed edges and any irregularities in structure are more susceptible to oxidation, so the edges appear more frayed (Figure 5b). The surface is undulating, and the resulting GO sheets are densely packed one on top of the other; thus, their two-dimensional structure is not easily visible. The obtained results are consistent with the literature data [56]. Modification with the use of epichlorohydrin causes the sheets to shrink and begin to delaminate. During the reaction, additional 2,3-epoxypropyl groups are introduced. Moreover, a polyether polymer can be formed on the surface of graphene oxide due to the high reactivity of epichlorohydrin. The introduced groups are bigger in size than hydroxyl groups, and the distance between adjacent graphene sheets increases. The modification promotes delamination of individual graphene sheets. The surfaces of the modified material are corrugated and very wrinkled (Figure 5c,d). More intensive treatment with longer contact time and higher epichlorohydrin dosage results in a more undulating surface.

Analyzing Figure 5d,f, it can be seen that the introduction of 2-hydroxymethyl-12-crown-4 onto the GO surface does not significantly affect its morphology, but the size of the resulting particles is smaller compared to GO modified only with epichlorohydrin. There is no distinctive difference between the material modified with 2-hydroxymethyl-12-crown-4 and hydroxy-DB14C4. The real microstructure of the obtained materials is disturbed in order to prepare the samples for SEM analysis. Characterization of graphene oxide-based materials using the SEM technique requires that they are previously centrifuged and dried, and thus graphene particles secondarily agglomerate (because of the drying of the material, the dried particles tend to curl due to van der Waals interactions between the graphene layers), which explains the large size of the obtained particles. Nevertheless, this does not reduce the suitability of the obtained materials as basic sorbents for lithium recovery.

#### 3.1.3. Structure Studies Using X-Ray Diffraction

**Figure 6:**
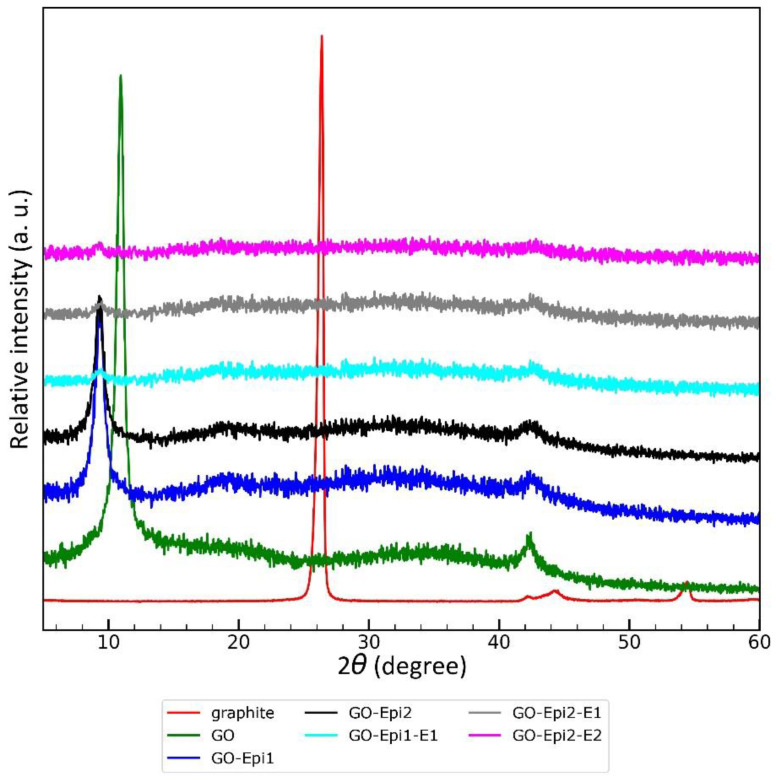
XRD patterns of raw graphite, graphene oxide (GO), GO modified with epichlorohydrin according to Procedure 1 (GO-Epi1), GO modified with epichlorohydrin according to Procedure 2 (GO-Epi2), GO modified with epichlorohydrin and 2-hydroxymethyl-12-crown-4 according to Procedure 1 (GO-Epi1-E1), GO modified with epichlorohydrin and 2-hydroxymethyl-12-crown-4 according to Procedure 2 (GO-Epi2-E1), GO modified with epichlorohydrin and hydroxy-DB14C4 (GO-Epi2-E2).

The XRD patterns are depicted in Figure 6. The main reflection in the diffraction pattern of the graphite used for synthesis is the (002) reflection at 2θ = 26.39° (FWHM = 0.451°), which corresponds to an interlayer 0.338 nm d-spacing. The (100) reflection was observed at 2θ = 42.26° (FWHM = 0.800°).

Graphite oxidation caused a visible change in the X-ray diffraction pattern. The (002) reflection present in graphite completely disappeared. The (001) reflection at 2θ = 10.966° is the most intense reflection in the graphene oxide XRD pattern, which is consistent with literature data [47]. The calculated interlayer d-spacing is equal to 0.806 nm, so the structure of the obtained material is less compact in comparison to graphite due to the introduction of oxygen-containing functional groups into the graphene planes and, consequently, a reduction in the interactions between the planes. The FWHM of (001) reflection (0.644°) is also higher than that of graphite because the crystallites of the obtained materials contain fewer graphene layers. It should be noted that the obtained material still contains multilayer graphene oxide. The (100) reflection of the obtained graphene oxide is still observed at approx. 42° (2θ = 42.314°); the shift during the oxidation is negligible, and the reflection does not broaden. Thus, it can be assumed that the size of graphene planes does not decrease. The results presented here are in good agreement with the SEM results discussed above and in the literature data [47].

The subsequent modification of graphene oxide with epichlorohydrin also causes changes in the XRD pattern. The (001) reflection shifts to lower angle values (2θ = 9.533° for GO-Epi1 and 2θ = 9.310° for GO-Epi2), and the reflection broadens (FWHM = 0.787° for GO-Epi1 and FWHM = 0.817° for GO-Epi2). Therefore, the introduction of 2,3-epoxypropyl groups causes a further increase in the distance between graphene layers. The corresponding d-spacings are equal to 0.927 nm for GO-Epi1 and 0.949 nm for GO-Epi2. This also indicates that the conditions for modification using epichlorohydrin are important for the structure of the obtained material. The position of the (100) reflection is still at approx. 2θ = 42.4°.

The grafting of crown ethers results in the almost complete disappearance of reflections; therefore, it can be assumed that the resulting material is highly disordered. This observation is also supported by the above-analyzed SEM results for ether-grafted graphene oxide. The separation and disorder of graphene planes is beneficial from the point of view of adsorption properties.

#### 3.1.4. Studies of Functional Groups Using Fourier Transform Infrared Spectroscopy

**Figure 7:**
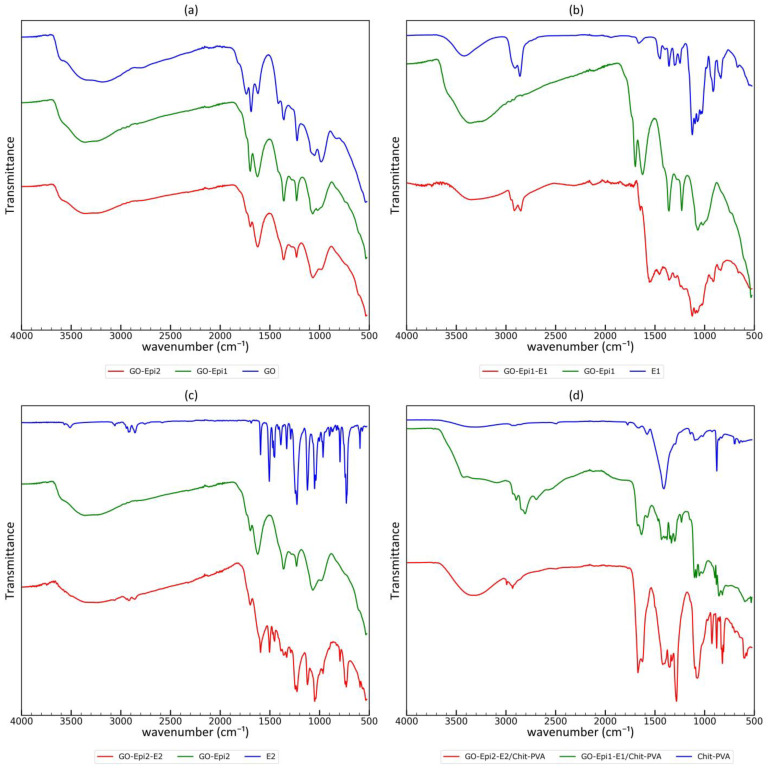
Comparison of FTIR spectra of materials at different stages of modification: (**a**) graphene oxide modification with epichlorohydrin; (**b**) 2-Hydroxymethyl-12-crown-4 grafting on the modified graphene oxide; (**c**) hydroxy-DB14C4 grafting on the modified graphene oxide; (**d**) comparison of prepared composite materials.

The characterization of functional groups of carbonaceous materials during modification was performed using ATR-FTIR spectroscopy; the corresponding spectra are shown in Figure 7. The spectrum of the prepared graphene oxide is similar to those reported in the literature [35,47,49]. Vibrations of different functional groups were identified in the material: O-H stretching associated with -OH (alcoholic and phenolic), -COOH and chemisorbed water (broad band 3000–3600 cm^−1^); C=O stretching associated with carbonyls such as quinone and ketone groups (1737 cm^−1^); C=O stretching of edge carboxyl groups (1695 cm^−1^) superimposed with water scissors vibrations; and stretching of sp^2^ hybridized C=C bonds of aromatic moieties (1617 cm^−1^). Two bands can be seen in the so-called γ-region [57] (with maxima at 1418 cm^−1^ and 1360 cm^−1^). According to the literature, such vibrations can be associated with epoxides, ethers, peroxides, m-benzoquinones, p-benzoquinones and ketones [57]. However, aliphatic C-H bending vibrations, as well as deformation vibrations of O-H groups (e.g., alcohol groups) can also occur in this range [58]. The subsequent strong band is observed at 1226 cm^−1^; according to the literature, this can be associated with C-O vibrations [47]. Several superimposed bands are visible in the so-called α and β regions [57] in the range from 900 cm^−1^ to 1180 cm^−1^. C-O-C stretching (symmetric and asymmetric) vibrations are responsible for the strong bands in this range. According to the literature [57], moieties such as lactols, peroxides, dioxolanes, hydroxyls, 1,3-dioxan-2-ones, anhydrides, carboxyls, epoxides oxolan-2-ones, ethers, ketones, pyran-2,3-diones, anhydrides, o-benzoquinones and p-benzoquinones are observed in this range. Therefore, when analyzing this region, it is difficult to use it to unambiguously assign bands to functional groups.

The comparison of the FTIR spectra for GO and graphene oxide reacted with epichlorohydrin clearly indicates that a new material has been obtained. The main changes in the FTIR spectra for graphene oxide exposed to the epichlorohydrin are observed in the following regions: (1) the same very weak bands appear in the range of 2880–3000 cm^−1^ and can be associated with C-H stretching of 2,3-epoxypropyl groups; (2) the band with a maximum at 1737 cm^−1^ disappeared (such an observation has also been reported in the literature [49]); (3) the band at 1690 cm^−1^ was probably shifted to 1700 cm^−1^ or to 1620 cm^−1^, and its relative intensity in the modified material is lower; (4) the relative intensity of the band at approx. 1620 cm^−1^ increased (the observed result may also be attributed to the overlap of another band shifted during the reaction); (5) the band at 1420 cm^−1^ in GO-Epi2 almost completely disappeared; and (6) significant changes can be seen in the shape of the bands in the so-called α and β regions in the range from 900 cm^−1^ to 1180 cm^−1^, which are similar to those reported in the literature [49], and the relative intensity of the band at 1073 cm^−1^ increases. As mentioned above, the discussion of the FTIR results of epichlorohydrin modification is difficult because other functional groups can be observed in the region associated with C-O-C vibrations characteristic for epoxy groups (900 cm^−1^–1180 cm^−1^). It can only be concluded that the number of fragments containing ether bonds increased as a result of epichlorohydrin modification. An interesting change in the FTIR spectra is the disappearance of bands at 1420 cm^−1^ (γ-region), as described above. This may be related to the reaction of -OH groups, which are present in graphene oxide. On the other hand, the disappearance of these bands rather excludes the hydrolysis of the epoxy groups introduced by the reaction of epichlorohydrin with graphene oxide. Moreover, the reaction of -OH groups of graphene oxide under basic conditions with epichlorohydrin through epoxy ring opening is also rather excluded, since in such a case 1 mole of an -OH group would be formed per 1 mole of reacted -OH groups derived from graphene oxide. Another interesting change is a clear increase in the intensity of the wide band with a maximum at 1620 cm^−1^. This is probably related to the partial rearomatization of the graphene planes and the formation of carboxylate anion groups (reaction with epichlorohydrin was carried out in the presence of NaOH). It should be mentioned here that quinone groups which are in keto-enol equilibrium are present in the structure of graphene oxide. During the reaction under basic conditions with epichlorohydrin, the keto-enol equilibrium of quinone moieties (containing C=O functional groups) shifts to the enol form (phenolic), which may react with epichlorohydrin. After the reaction, an aromatic–aliphatic ether bond is formed, and the ring is rearomatized. Such a mechanism corresponds well to both the disappearance of the band at 1737 cm^−1^ and the increase in the intensity of the band at 1620 cm^−1^.

In the next step, the crown ethers were reacted with modified graphene oxide. Characteristic bands associated with the crown ethers appear in the materials after modification (see Figure 7b,c), indicating that the crown ethers were bonded to the material. The main difference in the two cases presented is the clearly visible bands in the 2800–3000 cm^−1^ range, which is associated with C-H stretching in the crown ether backbone. A broad, strong and complex band in the range of 960–1208 cm^−1^ observed in pure 2-Hydroxymethyl-12-crown-4, probably associated with C-O-C symmetric and asymmetric stretching, is also observed in the modified material. However, in this case, several other functional groups also absorb in this region, so it cannot be used as a clear evidence of crown ether bonding. Two other bands associated with hydroxymethyl-12-crown-4 observed at 837 and 912 cm^−1^ are a better diagnostic tool, as only the α band edge is present in this region in the spectrum of modified graphene oxide (GO-Epi). The presence of such bands in the synthesized material clearly indicates the anchoring of the crown ether. A similar situation is observed in the case of hydroxy-DB14C4. A good diagnostic tool for evaluating crown ether bonding is the band at 729 cm^−1^, which is probably associated with the C-H out-of-plane deformation vibration of the aromatic rings of the crown ether.

In the last step, composite materials based on PVA–chitosan hydrogel filled with crown ether-grafted graphene oxides were prepared. The FTIR spectra of such materials are complex, but it should be noted that there are significant differences between the unfilled PVA–chitosan hydrogel and the filled samples. The main observed bands in the unfilled PVA–chitosan hydrogel are 3350 cm^−1^ O-H and N-H stretching of alcohol groups in PVA and amine groups in chitosan; 2800–3000 cm^−1^ aliphatic C-H stretching; 1670 cm^−1^ C=O stretching of amide bonds in chitosan (residual acetic amide groups); 1415 cm^−1^ C-H bending vibrations of PVA skeleton; and a complex band at 960–1180 cm^−1^ associated with C-O bonds occurring in chitosan as well as in PVA. After filling the composite with crown ether-grafted graphene oxides, several additional bands could be observed: strong bands in the range of 1550–1770 cm^−1^ that are associated with carboxylic and carbonyl groups in graphene oxide, two bands at 879 and 900 cm^−1^ that are characteristic of hydroxymethyl-12-crown-4 (slightly shifted in comparison to neat crown ether) and strong bands in the range of 900 cm^−1^ to 1180 cm^−1^, which are associated with ether groups.

To sum up, the analysis of the FTIR spectra of the materials obtained after each step of preparation provides a lot of valuable information on the structure of the resulting material. Taking into account the XRD and SEM results, as well as literature data [35,49] regarding similar materials, it can be concluded that the crown ethers were successfully incorporated into the composite. Moreover, to the best of our knowledge, this is the first attempt to incorporate dibenzo-14-crown-4 into a chitosan–PVA composite that exhibits better affinity for lithium cations than the commercially available hydroxymethyl-12-crown-4.

#### 3.1.5. Studies of Elemental Composition Using Energy-Dispersive X-Ray Spectroscopy

In light of the discussion presented above on changes in the properties of materials studied by SEM, XRD and FTIR, as well as the doubts and challenges with the interpretation of the results obtained, we decided to perform additional analyses for the novel crown ether-grafted graphene oxide material (GO-Epi2-E2) and for intermediate products used for its synthesis (GO, GO-Epi2).

Elemental composition was determined using the EDS technique at two different acceleration voltages: 5 kV and 15 kV. This approach enables the comparison of the elemental compositions of the sample at different penetration depths, facilitating the discussion of sample homogeneity, among other aspects. Results are presented in Table 2.

Phosphorus, sulfur, manganese and chlorine were detected only in the sample of graphene oxide and only at 15 kV. This indicates that the purification process of graphene oxide was carried out properly, with reagents used during synthesis (H_2_SO_4_, H_3_PO_4_, KMnO_4_ and HCl) being effectively washed away, and their residues remained only in the pores of the material (see results for 15 kV). Furthermore, the product obtained from the reaction with epichlorohydrin did not contain chlorine atoms, thereby excluding the presence of chlorohydrin moieties in the material’s structure. Both GO-Epi2 and GO-Epi2-E2 contain significant amounts of sodium, which is associated with the presence of sodium salts of carboxylic groups in the structure of these materials. NaOH was used as the base in the synthesis of GO-Epi2, while NaH was used in the synthesis of GO-Epi2-E2. The synthesis products were washed with water but not exposed to acidic solution, so the modified graphene oxides contain carboxylic groups in the form of sodium salts.

Since hydrogen atoms cannot be detected using the EDS method, it is more advantageous to further analyze the variability in the C/O and Na/O atomic ratios (presented in the Table 3). The C/O ratio is widely used in the literature [47,58] to compare different graphene materials and is associated with the so-called “level of oxidation” of graphene planes [47], which is a measure of the oxygen containing groups introduced to the material. The Na/O ratio is related to the content of carboxyl groups in the form of sodium salts in the structure of the synthesized materials.

The synthesized GO is characterized by a C/O ratio of 1.27–1.28, which is lower than the values reported in the literature [39], which typically range from 1.85 to 2.3. A high C/O ratio is advantageous for the synthesis of adsorbents (more oxygen containing functional groups are available for further modification). It should be noted that the C/O ratio calculated on the basis of the EDS results mainly concerns the surface layer of the studied material.

The C/O ratio increased significantly after the reaction with epichlorohydrin, rising from an average of 1.3 to 2.6, and then slightly increased to 2.8 after the introduction of crown ether. Consequently, the relative oxygen content in the material decreased substantially following the reaction with epichlorohydrin. The occurrence of only the reaction of NaOH with GO, resulting in the formation of a material rich in fragments of sodium salts of carboxylic acids, cannot change the C/O ratio, so the reaction must have resulted in the functionalization of GO. The introduction of 2,3-epoxypropyl groups via the reaction of epichlorohydrin with the epoxy groups (activated by base) of graphene oxide leads to an increase in the C/O ratio. Since the C/O ratio for a 2,3-epoxypropyl unit is equal to 3, the formation of modified GO material with C/O = 2.6 is allowed. Possible (and reported in the literature) subsequent polymerization [52] (Figure 2b) of 2,3-epoxypropyl groups introduced into the GO material with an excess of epichlorohydrin results in the formation of a material characterized by a C/O ratio of approx. 1.5. Thus, this reaction may only be of secondary importance in the discussed case.

To sum up, such a high increase in the C/O ratio is possible only if 2,3-epoxypropyl groups are introduced into the material.

The reaction with crown ether slightly altered the C/O ratio (for both 5 and 15 kV results), which is consistent with the C/O ratio of crown ether units introduced into the material. The C/O ratio for hydroxy-DB14C4 is equal to 3.6.

It is important to note that altering the acceleration voltage, and thus the penetration depth during EDS measurements, results in changes to the composition of the modified GO. Deeper layers of the material exhibit lower C/O and Na/O ratios, suggesting they are less modified, the concentrations of introduced 2,3-epoxypropyl groups and crown ether units are slightly lower.

The significant amount of sodium (5–7 atomic%) in the modified graphene oxides indicates a relatively high ion exchange capacity of the obtained materials.

#### 3.1.6. Aromatization Level Determined by Raman Spectroscopy

**Figure 8:**
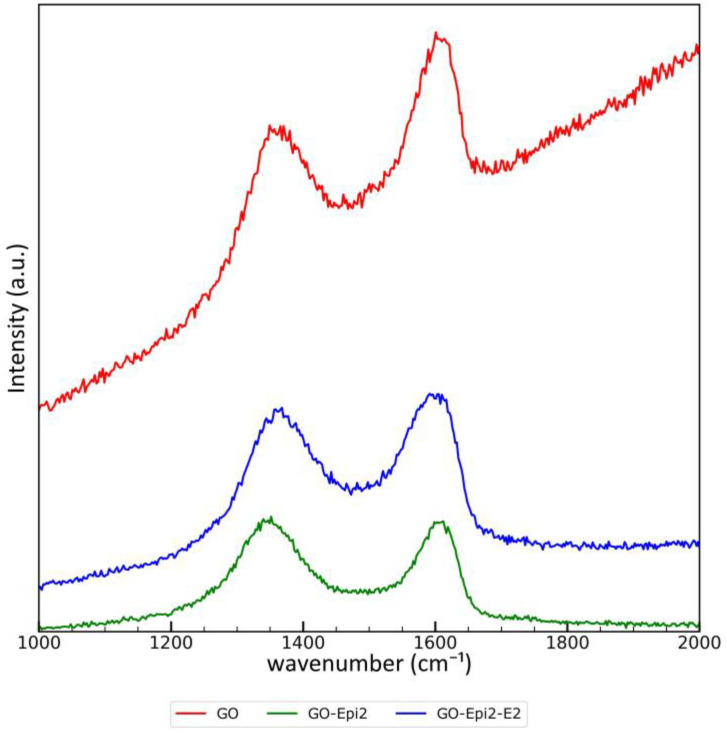
Comparison of Raman spectra of selected materials.

Given that the modification of GO with epichlorohydrin can lead to the rearomatization of graphene planes (see discussion of FTIR results), we decided to study selected materials (GO, GO-Epi2 and GO-Epi2-E2) using Raman spectroscopy. The recorded spectra are presented in Figure 8.

The high background observed for graphene oxide is due to its fluorescence in the near-infrared region (500–800 nm) [59]. After modification with epichlorohydrin, the fluorescence was minimized. Two typical bands (for graphene-based materials) [58] are observed:

1. The D band, with a maximum at 1361 cm^−1^ for GO, 1345 cm^−1^ for GO-Epi2 and 1363 cm^−1^ for GO-Epi2-E2;
2. The G band, with a maximum at 1605 cm^−1^ for GO, GO-Epi2 and GO-Epi2-E2.

The position of the D band, associated with disordered regions having sp^3^ carbons connected to out-of-plane vibrations, is slightly dependent on the modification stage of the material. In contrast, the position of the G band, associated with in-plane vibrations of sp^2^ carbons, is virtually the same for all materials. The reaction of graphene oxide with epichlorohydrin and subsequently with crown ether alters the surroundings of sp^3^ carbons in the graphene plane, so the position of the D band is more sensitive to such modification (due to the proximity of modified sites) than the G band.

The intensity ratio of the D to G_app_ (superimposed G and D’ bands) bands (I_D_/I_Gapp_) [60] is a relative measure of the average size of the sp^2^ network and is commonly used in characterizing of graphene-based materials. The calculated I_D_/I_Gapp_ ratios are: 0.86 for synthesized GO, 1.14 for GO-Epi2, and 0.94 for GO-Epi2-E2. The typical range for I_D_/I_Gapp_ for graphene oxide synthesized by oxidation using KMnO_4_ is in the range of 0.69–0.98 [58]. The increase in I_D_/I_Gapp_ after modification of GO via the reaction of epoxy groups is frequently reported in the literature (e.g., [38]).

Crown ether molecules exhibit band active in Raman mode in the range of 1250–1300 cm^−1^ and 1450–1500 cm^−1^. These bands are covered by broad D and G bands associated with the GO structure, so bands related to the crown ether moiety cannot be observed [43].

### 3.2. Adsorption Properties of the Studied Materials

Static adsorption tests were carried out on the materials obtained at each stage of the synthesis in order to quickly assess the affinity for lithium ions. Two series of experiments were performed, the first using pure lithium chloride solution with a lithium concentration of 200 mg/kg and the second with real brine containing approx. 200 mg/kg of lithium ions. Here, it is important to emphasize that direct lithium extraction methods are being developed for so-called secondary lithium sources, like geothermal brines, produced water from oil and gas fields, etc. According to the literature data, the concentration of lithium in such media is in the range of 0.038–505 mg/L for oilfield brines [61] and 0.3–440 mg/L for geothermal brines [32]. Therefore, the use of a solution containing 200 mg/kg of lithium ions seems to be reasonable. Adsorption experiments were carried out under similar conditions as in our other comparative study focusing on lithium adsorption on different materials [31], so the data presented can be easily compared.

The results presented in Table 4 include the distribution coefficient, recovery and operating capacity measured at pH 5 and pH 8 for various materials contacted with the brines at a solution to material mass ratio equal to 10/1. The distribution coefficient is a better parameter for assessing the affinity of material for lithium ions than the maximum adsorption capacity, because the maximum adsorption capacity is typically determined at high lithium concentration and high solution to sorbent mass ratio. The adsorbent works under much worse conditions in cases closer to reality, e.g., when the feed concentration is lower, contact times are limited, etc. It should be added that, according to the literature data [35], crown ether-modified materials operate most efficiently at pH 8, while the pH of the raw brine was slightly acidic; therefore, we chose the aforementioned pH values for adsorption experiments.

The most impressive results reported in the literature [35] indicate that the PVA–chitosan composite material filled with crown ether-grafted graphene oxide exhibits a high lithium capacity that is superior to lithium ion sieves. However, the cited results were determined with the use of a highly concentrated solution (1000 mg/L pure lithium chloride solution). Moreover, the value 168.5 mg/g is the maximum lithium capacity according to the Langmuir model. For this reason, we decided to study such material at a lower lithium concentration and using a complex brine. The results of the operating capacity for the material prepared according to the literature at a low lithium concentration (feed 200 mg/kg; equilibrium 75 mg/kg), shown in Table 4, indicate that the material has a lithium capacity of 2.8 mg/g in the case of pure LiCl solution, and 1.6 mg/g in the case of complex brine. Therefore, in our opinion, comparing different lithium capacities in a single table without commenting on the experimental conditions (e.g., as in reference [5]) can lead to misunderstandings. The second issue is the selectivity of the prepared material—according to the literature [35], PVA–chitosan composite material filled with crown ether-grafted graphene oxide exhibits a selectivity coefficient ranging from 2.27 (for Mg^2+^) to 4.19 (for K^+^). These data were determined using brine containing a similar (to the lithium ion) concentration of interfering ions. Concentrations of such ions as Na^+^, Mg^2+^, K^+^ and Ca^2+^ in real samples of brine are at least an order of magnitude higher. Therefore, the affinity for lithium of all the materials we tested decreases by a factor of 10–100. This is also quite an obvious conclusion considering the selectivity coefficients presented in the literature [35]. To sum up, these types of composite materials may have some advantages for poorly saline lithium-rich media (e.g., in the concentration of DLE eluates [6]), but such materials are of limited use for highly saline brines. However, it should be noted that under slightly acidic conditions (pH 5), several of the obtained materials exhibit a similar distribution coefficient for complex brine as the previously tested lithium ion sieves (lithium manganese oxide and lithium titanium oxide): 0.8–1.5 for the currently tested materials and approx. 2 for the previously tested γ-Al_2_O_3_ lithium manganese oxides (samples named as LIS10-LIS12), as well as lithium titanium oxides (samples named as LIS3,LIS4) [31].

Taking a closer look at the results in Table 4 for the materials obtained after different synthesis stages, it can be seen that in pure lithium chloride solution, even graphene oxide has moderate affinity for lithium ions that is comparable to commercially available mineral adsorbents (including zeolites), organophosphate extractants, as well as carbon-based adsorbents. This is due to the presence of carboxylic functional groups in the graphene oxide structure. Thus, this material should be considered a weak acidic general-purpose ion exchanger, which is why almost all the affinity for lithium ions observed in pure solution disappears in the case of complex brine. Modification of GO with epichlorohydrin increases the affinity for lithium ions: GO-Epi1 and GO-Epi2 exhibit comparable affinity to some commercially available ion exchange resins in pure solution. Modification of graphene oxide with epichlorohydrin, as discussed above, may result in an increase in the distance between graphene planes and, consequently, greater availability of carboxylic functional groups. Moreover, polyether moieties may also interact with lithium ions, as recently reported in the literature [62].

The distribution coefficients for 2-Hydroxymethyl-12-crown-4 and E2 were also measured according to the procedure described in [31]. It is important to note that K_d_ for the pure lithium chloride solution is lower than for the brine. This is probably due to the moderate solubility in aqueous solution of this crown ether, so the extractant also is partitioned between the organic and aqueous phase. In a complex, almost saturated brine, the solubility of crown ether is lower, so K_d_ is also increased. The reported K_d_ values are similar to those for commercially available organophosphate extractants such as tributyl phosphate (TBP) or bis(2-ethylhexyl) hydrogen phosphate (D2EHPA) [31], and much higher in complex brines. The determined K_d_ values for crown ether are much lower than those reported in the literature for different lithium selective crown ethers [50,54], but it should be emphasized that lithium perchlorate is typically used as a lithium source in extraction experiments [54]. It is known that the affinity of perchlorates of alkali metals for the organic phase is much higher than for chlorides because of the solvation energy and weak coordination properties of the perchlorate anion [63]. Therefore, such results overestimate the affinity of crown ether solutions in real chloride brines. To the best of our knowledge, there is a lack of data on distribution coefficients in complex brine systems. Here, we reported two K_d_ values: 1.7 and 1.9 for 2-Hydroxymethyl-12-crown-4 and hydroxy-DB14C4 extractants measured in complex brine. These values are significantly higher than those we measured for other commercially available extractants that exhibit negligible extraction efficiency in complex brine [31].

Analysis of the results for chitosan–PVA composites leads to several conclusions. Unfilled Chit-PVA composite exhibits some non-negligible affinity for lithium ions in both pure lithium chloride solution and complex brine. This is probably the effect of an interaction with hydroxyl and acetal groups. The possibility of such an interaction has recently been discussed in the literature [62]. The introduction of crown ether-grafted graphene oxide into the composite structure causes an increase in the distribution coefficient in both systems (pure lithium chloride solution and complex brine). Comparison of filled composites with modified graphene oxides with crown ethers indicates that the distribution coefficient in the pure LiCl solution is greatly reduced, while in the complex brine, the opposite trend is observed (K_d_ values increase slightly). The first effect may be explained by the dilution of the active material by the composite matrix, as there are fewer adsorption functional groups (carboxylic, as well as lithium-selective crown ether and polyether moieties). The second trend indicates that there may be some synergistic effect between the functional groups of the composite matrix and the active material.

Comparison of the presented results with literature data [35] leads to several final remarks: (1) the main factor responsible for the adsorption of lithium in the pure system is graphene oxide (probably the carboxyl groups of the material), and the impressive maximum capacity of the material is associated with the high content of non-selective carboxylic groups (please see Figure 8 in [35]—in a certain range of lithium concentrations, composites containing unmodified graphene oxide exhibit higher capacity than those modified with crown ether); (2) the second argument in favor of the above conclusion is the value of the adsorption capacity of the material reported in [35], which is equal to 168.50 mg/g and is much higher than the maximum capacity of the pure 12-crown-4-ether molecule (the maximum capacity is equal to 39.3 mg/g, assuming the formation of a 1:1 complex of lithium cation and crown ether; in the case of a 2:1 complex, the maximum capacity is lower), and therefore most of the reported capacity must be associated with graphene oxide; (3) the third argument supporting the above conclusion is the small difference between the maximum capacity of the modified with crown ether and unmodified material described in [35]—the difference is approx. 20 mg/g and may be associated with lithium-selective groups in the reported material; (4) taking into account such a corrected value of the maximum capacity of the lithium selective group, the results obtained by us at a significantly lower lithium concentration and a higher concentration of interfering ions corresponds fairly well with literature data [35]; and (5) the application of more selective hydroxy-DB14C4 increases the affinity of the composite material.

Preliminary trials for the regeneration of two materials, GO-Epi2-E1/Chit-PVA and GO-Epi1-E2/Chit-PVA, were also performed using 0.01 M HCl as a regenerant. Based on preliminary calculations, the recovery was >67%.

## 4. Conclusions

Three composite materials based on polyvinyl alcohol–chitosan hydrogel containing crown ether-grafted graphene oxide were successfully synthesized and tested in pure diluted lithium chloride solution, as well as in real complex brine. It was shown that the distribution coefficient values for the obtained composites in real brine in a slightly acidic solution are similar to the values determined previously for lithium-selective sieves under similar conditions. Moreover, the intermediate products’ affinity for lithium ions was characterized. The study shows that the affinity for lithium in pure LiCl solution is mostly associated with non-selective interaction of lithium ions with graphene oxide matrix (COOH groups). The results were compared with literature data; after a detailed discussion, it was found that the results presented are fairly consistent with previously reported data. Crown ether-modified materials exhibit good affinity for lithium ions in pure LiCl solution (K_d_ values up to 75.6) and may be used to recover lithium from low saline waters, but their application is limited for highly saline complex oilfield brines (K_d_ values up to 2.3). It was also shown that the application of dibenzo-14-crown-4 moiety to graphene oxide modification groups increases the affinity of the composite material for lithium ions compared to the analogous material containing 12-crown-4-ether groups. Therefore, we believe it is possible to develop more selective crown ether that contains composite materials for lithium recovery from complex, highly saline brines, and our work provides valuable guidance in this regard.

## Figures and Tables

**Table 1:** Description of the obtained materials.

Sample Name	Description
Chit-PVA	chitosan–PVA composite
GO-Epi1-E1/Chit-PVA	chitosan–PVA composite with addition of GO-Epi1-E1 material
GO-Epi2-E1/Chit-PVA	chitosan–PVA composite with addition of GO-Epi2-E1 material
GO-Epi2-E2/Chit-PVA	chitosan–PVA composite with addition of GO-Epi2-E2 material

**Table 2:** EDS results for selected samples recorded at two different acceleration voltages: 5 kV and 15 kV. Results for 15 kV are presented in brackets.

Sample Name	Atomic Percent						
	C	O	Mn	Na	Cl	S	P
GO	56.1 (55.3)	43.9 (43.3)	0.0 (0.0)	0.0 (0.0)	0.0 (0.2)	0.0 (1.1)	0.0 (0.1)
GO-Epi2	69.2 (66.6)	23.9 (28.0)	0.0 (0.0)	6.9 (5.4)	0.0 (0.0)	0.0 (0.0)	0.0 (0.0)
GO-Epi2-E2	68.6 (68.6)	25.6 (26.6)	0.0 (0.0)	5.8 (4.8)	0.0 (0.0)	0.0 (0.0)	0.0 (0.0)

**Table 3:** C/O and C/Na ratios calculated based on the EDS results. Results for 15 kV are presented in brackets.

Sample Name	Atomic Ratio	
	C/O	Na/O
GO	1.28 (1.27)	0.00 (0.00)
GO-Epi2	2.90 (2.38)	0.29 (0.19)
GO-Epi2-E2	2.93 (2.58)	0.23 (0.18)

**Table 4:** Results of adsorption tests.

Sample Name	K_d_ Li * (K_d_ Li pH 8)	K_d_ Brine ** (K_d_ Brine pH 8)	R Li * (R Li pH 8)	R Brine ** (R Brine pH 8)	q Li * (q Li pH 8)	q Brine ** (q Brine pH 8)
GO	7.2 (15.2)	0.2 (0.4)	28% (45%)	2% (4%)	0.89 (1.47)	0.05 (0.08)
GO-Epi1	26.9 (11.6)	0.5 (0.3)	54% (34%)	6% (3%)	2.08 (1.34)	0.11 (0.05)
GO-Epi2	46.4 (25.2)	0.8 (0.2)	75% (62%)	6% (2%)	1.96 (1.67)	0.17 (0.05)
E1	0.9 (2.5)	1.7 (1.2)	3% (7%)	11% (8%)	0.16 (0.41)	0.32 (0.24)
E2	1.2 (1.4)	1.9 (2.0)	5% (6%)	8% (9%)	0.20 (0.24)	0.40 (0.42)
GO-Epi1-E1	17.0 (13.3)	0.8 (1.3)	67% (64%)	12% (19%)	0.89 (0.86)	0.15 (0.24)
GO-Epi2-E1	22.1 (15.3)	1.2 (1.3)	68% (63%)	16% (18%)	1.11 (1.02)	0.22 (0.25)
GO-Epi2-E2	75.6 (60.3)	1.2 (1.7)	78% (76%)	17% (21%)	2.64 (2.62)	0.23 (0.32)
Chit-PVA	0.8 (0.9)	1.1 (1.0)	4% (4%)	6% (6%)	0.13 (0.16)	0.25 (0.23)
GO-Epi1-E1/Chit-PVA	2.8 (3.0)	1.6 (1.7)	21% (22%)	14% (15%)	0.40 (0.41)	0.32 (0.34)
GO-Epi2-E1/Chit-PVA	2.9 (3.2)	1.8 (1.9)	22% (23%)	16% (16%)	0.40 (0.44)	0.35 (0.37)
GO-Epi1-E2/Chit-PVA	8.2 (8.7)	2.2 (2.3)	35% (37%)	16% (16%)	0.95 (0.98)	0.43 (0.45)

* Distribution coefficient, recovery (%) and operation capacity [mg/g] determined in pure LiCl solution at pH approx. 5; the values determined at pH 8 are given in brackets. ** Distribution coefficient, recovery (%) and operation capacity [mg/g] determined in real brine sample at pH of approx. 5; the values determined at pH 8 are given in brackets.

## Data Availability

The original contributions presented in this study are included in the article. Further inquiries can be directed to the corresponding author.
